# Multistep organic synthesis of modular photosystems

**DOI:** 10.3762/bjoc.8.102

**Published:** 2012-06-19

**Authors:** Naomi Sakai, Stefan Matile

**Affiliations:** 1 Department of Organic Chemistry, University of Geneva, Geneva, Switzerland

**Keywords:** asparagusic acid, charge-transfer cascades, chromophores, disulfide exchange, hydrazone exchange, molecular switches, naphthalenediimides, π-stacks, surface-initiated polymerization

## Abstract

Quite extensive synthetic achievements vanish in the online supporting information of publications on functional systems. Underappreciated, their value is recognized by experts only. As an example, we here focus in on the recent synthesis of multicomponent photosystems with antiparallel charge-transfer cascades in co-axial hole- and electron-transporting channels. The synthetic steps are described one-by-one, starting with commercial starting materials and moving on to key intermediates, such as asparagusic acid, an intriguing natural product, as well as diphosphonate “feet”, and panchromatic naphthalenediimides (NDIs), to finally reach the target molecules. These products are initiators and propagators for self-organizing surface-initiated polymerization (SOSIP), a new method introduced to secure facile access to complex architectures. Chemoorthogonal to the ring-opening disulfide exchange used for SOSIP, hydrazone exchange is then introduced to achieve stack exchange, which is a “switching” technology invented to drill giant holes into SOSIP architectures and fill them with functional π-stacks of free choice.

## Introduction

**Figure 1:**
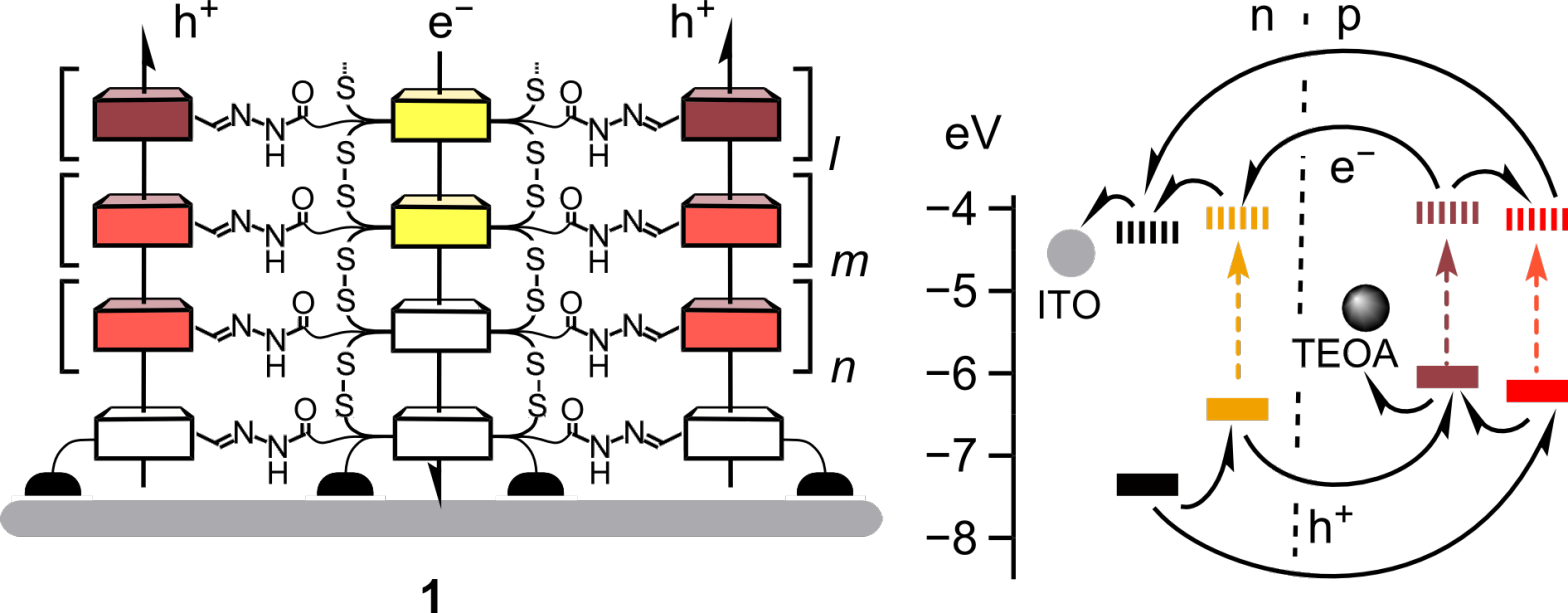
Schematic structure of photosystem **1** on indium tin oxide (ITO, grey) with antiparallel gradients in hole (p, h^+^) and electron (n, e^−^) transporting coaxial channels. HOMO (solid) and LUMO levels (dashed) of all components (color coded) are indicated in eV against vacuum (−5.1 eV for Fc/Fc^+^), including triethanolamine (TEOA), used as mobile hole transporter.

The architecture of photosystem **1** is rather sophisticated, probably as sophisticated as it gets with photosystems today (Figure 1) [1]. It is composed of three co-axial π-stacks that are grown from an indium tin oxide (ITO) surface. With lower frontier molecular orbital (FMO) levels, the “yellow” stacks can transport photogenerated electrons toward the ITO surface along the gradient in their LUMO. With higher FMO levels, the “red” stacks can transport holes along the gradient in their HOMOs in the opposite direction, away from the ITO surface.

The double-channel architecture **1** with antiparallel redox gradients has been referred to as OMARG-SHJ, that is supramolecular n/p-heterojunctions with oriented multicomponent/color antiparallel redox gradients [2–3]. With n/p contact areas maximized down to the molecular level, photoinduced charge separation (i.e., charge generation) should be as favorable as directional charge translocation (i.e., charge separation) along redox gradients in the molecular channels. With photosystem **1**, these high expectations could finally be tested experimentally [1]. OMARG-SHJs turned out to be best quantified with bimolecular charge recombination efficiencies η_BR_, that is, losses in photonic energy. Photosystem **1** gave η_BR_ = 22%; gradient-free controls gave η_BR_ = 50%; destructive gradients gave η_BR_ = 76%. These results are very satisfactory. They also confirmed that significant synthetic efforts to build sophisticated functional architectures can be worthwhile. In the original communication, these synthetic efforts completely disappeared in the online supporting information [1]. The fact that results on synthesis are self-explanatory to all and do not require much discussion can be considered as a marvelous illustration of the success of the field. However, to illustrate the frequent lack of appreciation of the synthetic organic chemistry in work on functional systems, the total synthesis of photosystem **1** will be described step-by-step in the following.

## Results and Discussion

### Synthesis of initiators

**Scheme 1:**
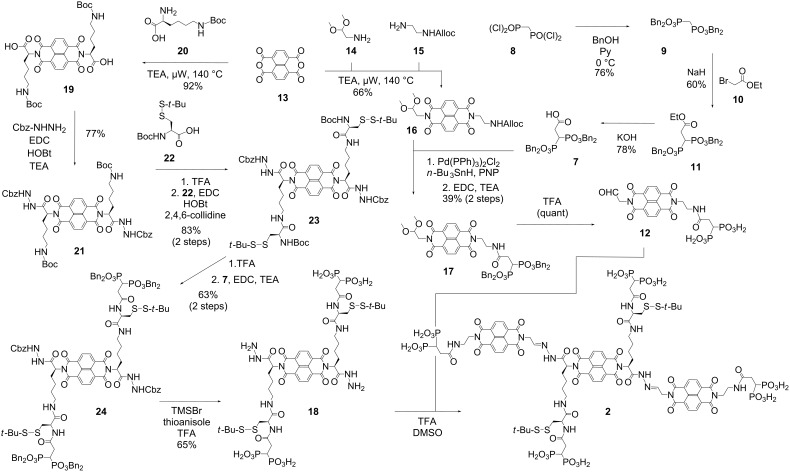
Synthesis of initiator **2**.

**Scheme 2:**
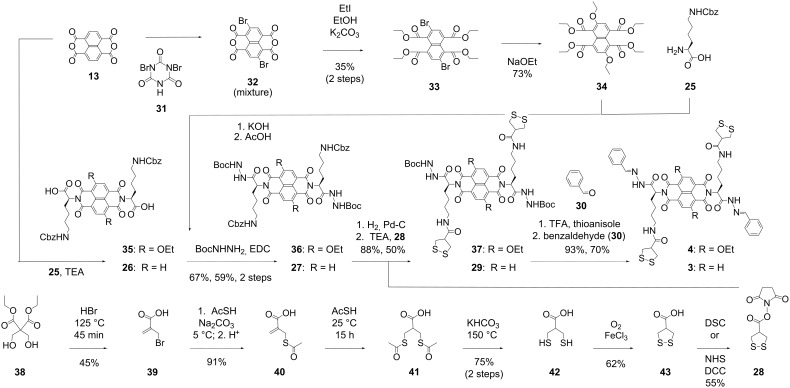
Synthesis of propagators **3** and **4**.

**Scheme 3:**
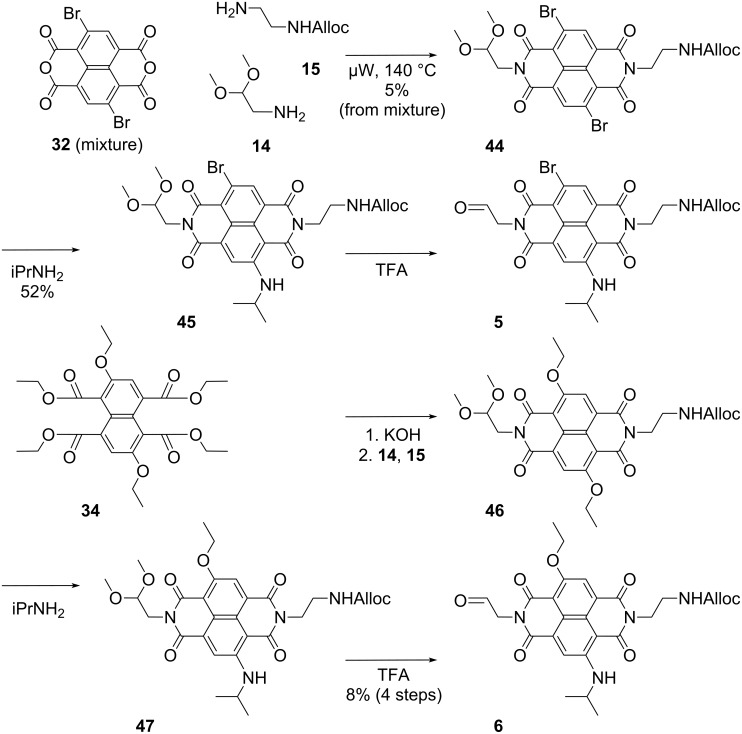
Synthesis of stack exchangers **5** and **6**. Compounds **5**, **6**, **45** and **47** are mixtures of 2,6- and 3,7-regioisomers.

Photosystem **1** is constructed from the molecular building blocks **2**–**6** (Schemes 1-3). Initiator **2** is composed of a central naphthalenediimide (NDI) [4–19] to act as a template for the central stack and two peripheral NDIs to act as templates for stack exchange. They are embedded into hydrogen-bonded networks, to assure self-organization, and four geminal diphosphonates [20–21] for tetravalent anchoring on the ITO surface.

The geminal diphosphonates **7** were synthesized from methylene bis(phosphonic dichloride) **8** (Scheme 1) [20–21]. Conversion with benzyl alcohol and pyridine as a base yielded tetrabenzyl methylene bisphosphonate **9**. Activation with sodium hydride and alkylation with ethyl bromoacetate (**10**) gave ethyl ester **11**, and final ester hydrolysis lead to the desired acid **7**.

The peripheral NDIs **12**, designed to template for stack exchange on initiator **2**, were prepared from naphthalenedianhydride (NDA) **13**. Microwave-assisted imidation [15] with the two amines **14** and **15** at 140 °C gave the mixed diimide **16** in excellent 66% yield together with the symmetric diimide side products. Amines were liberated by palladium-catalyzed Alloc removal and reacted with acid **7**. The obtained amide **17** was deprotected with acid to afford the desired aldehyde **12**.

The central NDI **18** of initiator **2**, designed to initiate and template for SOSIP, was accessible from NDA **13** as well. The synthesis of NDI **19** by microwave-assisted imidation with Boc-protected lysine **20** has been reported before in the literature [15]. Reaction with Cbz-hydrazine gave the Cbz-protected NDI hydrazide **21**. After chemoselective, acid-catalyzed deprotection, the liberated amines were coupled with the Boc-protected cysteine *tert*-butyl disulfide **22**. The obtained amide **23** was treated with TFA for Boc removal and coupled with the geminal diphosphonate foot **7**. Deprotection of both hydrazides and diphosphonates in NDI **24** gave **18**, which was reacted in situ with NDI **12** to yield initiator **2**.

### Synthesis of propagators

The synthesis of propagator **3** starts with NDA **13** as well (Scheme 2). Diimidation with Cbz-protected lysine **25** gave the diacid **26**. Activation with EDC, HOBt and TEA was followed by the reaction with *tert*-butyl carbazate under mild conditions. The protected hydrazide **27** was obtained in 59% yield over two steps. The Cbz protecting groups were removed chemoselectively by hydrogenolysis over Pd–C in the presence of acetic acid, and the obtained diamine was reacted with the activated asparagusic acid **28**. Hydrazide deprotection in NDI **29** and in situ hydrazone formation with benzaldehyde **30** gave propagator **3**.

In contrast to propagator **3**, propagator **4** is constructed around a yellow, core-substituted cNDI fluorophore. Nevertheless, the synthesis of this target molecule also starts with NDA **13**. Bromination in the core with dibromocyanuric acid (**31**) afforded an intractable mixture containing the 2,6-dibromo NDA **32** together with lower and higher homologues [16]. However, pure product **33** could be readily isolated from this mixture after transformation of the NDAs into the core-substituted naphthalenetetraesters (cNTEs). Nucleophilic core-substitution with ethanolate gave cNTE **34** as described in the literature [16].

NTE **34** was subjected to basic ester hydrolysis followed by diimidation with lysine **25**. From this point, the synthesis of cNDI propagator **4** was analogous to the synthesis of NDI propagator **3**. Reaction of EDC-activated diacid **35** with *tert*-butyl carbazate followed by deprotection of the obtained cNDI **36** and coupling with activated asparagusic acid **28** gave cNDI **37**. Hydrazide deprotection quenched by benzaldehyde **30** gave the yellow cNDI propagator **4**.

The activated asparagusic acid **28** was prepared by following literature procedures [22–25]. In the first step from bis(hydroxymethyl)malonate **38**, simple nucleophilic substitution is coupled with an ester hydrolysis and a debrominative decarboxylation. Another nucleophilic substitution with thioacetate converted bromide **39** into thioester **40**. Addition of a second thioacetate gave dithioester **41**, which was hydrolyzed with a base. Oxidation of dithiol **42** with molecular oxygen gave asparagusic acid (**43**), which is the natural product that contributes to the characteristic odor of asparagus. Activation with NHS gave the ester **28**, ready for coupling with amines, such as **27** or **36**.

### Synthesis of stack exchangers

The synthesis of the red cNDIs **5** and **6** for stack exchange was possible in very few steps starting from available synthetic intermediates (Scheme 3). cNDI **5**, with one bromo and one alkylamino substituent in the core, was prepared from crude dibromo cNDA **32**. Microwave-assisted reaction [15] with amines **14** and **15** gave the mixed cNDI **44** together with the symmetric side products. The obtained mixture of 2,6- and 3,7-regioisomers was not separated throughout the entire synthesis of photosystem **1**. Nucleophilic aromatic substitution with isopropylamine for 10 min at room temperature gave the red cNDI **45**, which was followed by deprotection with acid to give the target aldehyde **5**.

The more pinkish cNDI **6** was synthesized from the cNTE **34** following the procedure developed for cNDI **5**. Diimide formation with amines **14** and **15** followed by core substitution of the mixed cNDI **46** and deprotection of the red cNDI **47** gave the desired aldehyde **6**.

### Self-organizing surface-initiated polymerization

**Scheme 4:**
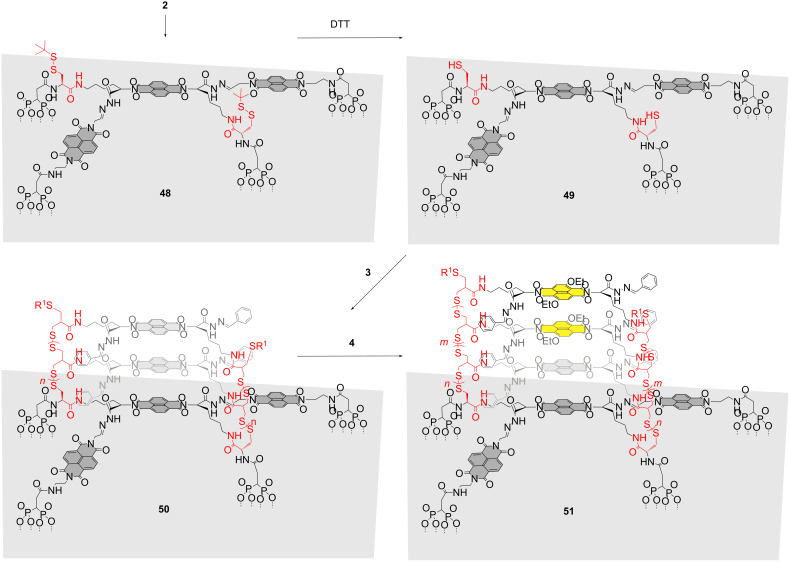
Synthesis of photosystem **1**, self-organizing surface-initiated polymerization (SOSIP). R^1^ = SH (**50**) or oxidized derivative (**51**), grey surface = ITO. Dotted lines from diphosphonate groups indicate the bonds to the ITO surface. The polymer structures are generalized and idealized structures, which are consistent with the experimental results. They will naturally contain defects.

With the five building blocks **2**–**6** in hand, the solid-phase synthesis of photosystem **1** on ITO surfaces could be launched. ITO was first cleaned with RCA solution, that is, a boiling 5:1:1 mixture of water, 24% NH_4_OH and 30% H_2_O_2_, and then rinsed with bidistilled water and EtOH, and dried. Then the ITO was immersed in a 3 mM solution of initiator **2** in DMSO for 2 days. The formation of monolayers of **48** on ITO electrodes was followed by the inhibition of potassium ferricyanide reduction in solution, and by absorption spectroscopy (Scheme 4). The obtained monolayers of **48** were annealed for 1 h in the oven at 120 °C. These conditions are known to improve the covalent bonding between phosphonic acids and the ITO substrate [26].

The disulfide protecting groups on the surface of monolayer **48** were removed with DTT to afford free thiols on the surface of monolayer **49**. For SOSIP [18–19], the concentration of propagators had to be optimized to a critical SOSIP concentration, *c*_SOSIP_. Below *c*_SOSIP_, ring-opening disulfide-exchange polymerization [27] does not occur, whereas above *c*_SOSIP_, the polymerization occurs everywhere, not only on the surface but also in solution. To determine *c*_SOSIP_, ITO plates with and without activated initiators were incubated together in the same solution of propagators. The amount of polymers either grown from the ITO surface or deposited on the ITO surface by precipitation during polymerization in solution was determined by absorption spectroscopy. Plots of the absorption of the electrode as a function of the concentration of the propagator in solution revealed both *c*_SOSIP_, the critical concentration needed for SOSIP, and *c*_SOL_, the critical concentration needed for polymerization in solution. Operational SOSIP was demonstrated with *c*_SOSIP_ < *c*_SOL_, and failure of SOSIP with *c*_SOSIP_ = *c*_SOL_. Both *c*_SOSIP_ and *c*_SOL_ depended strongly on the conditions, i.e., the concentration and nature of the base catalyst, the nature of initiator and propagator, the temperature, the presence of oxygen in the solution and, most importantly, the composition of the solvent mixture used.

For propagator **3**, *c*_SOSIP_ = 3.5 mM was found in a 1:1 mixture of chloroform and methanol with 100 mM DIPEA as a base catalyst. Incubation of monolayer **49** in this solution gave SOSIP architecture **50**. To add the yellow stacks in photosystem **1**, photosystem **50** was incubated with propagator **4** at *c*_SOSIP_ = 7 mM in chloroform/methanol (1:1) with 100 mM DIPEA. The obtained oriented diblock disulfide polymers **51** were characterized by the absorption of colorless NDIs at 385 nm and the absorption of yellow cNDIs at 470 nm. Assuming regular growth, these absorptions provided a meaningful approximation of the average composition *n* and *m* of the poly(disulfide) [27].

### Stack exchange

**Scheme 5:**
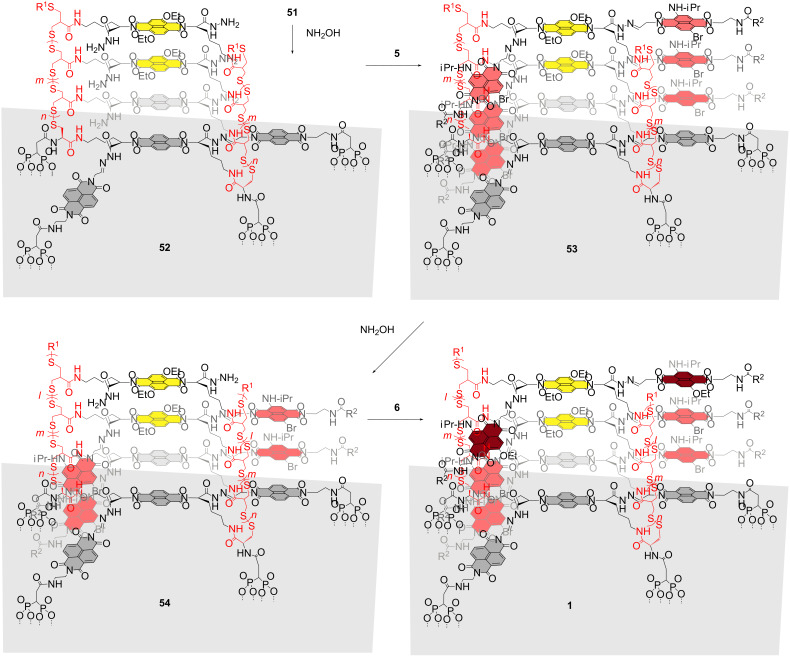
Synthesis of photosystem **1**, stack exchange. R^1^ = SH or oxidized derivative, R^1^ = CH_2_CHCH_2_. **5** and **6** are mixtures of 2,6- and 3,7-regioisomers.

Stack exchange within the resulting SOSIP photosystem **51** was initiated with excess hydroxylamine (Scheme 5). The chemoorthogonality of disulfide and hydrazone exchange has been demonstrated previously by several groups [28–31]. Benzaldehyde removal as oxime was followed by HPLC. The hydrazide-rich pores produced in the resulting architecture **52** were first filled by reversible covalent capture of the red cNDI aldehyde **5**.

Stack exchange was easily detectable by comparison of the respective maxima in the absorption spectra. Exchange of the benzaldehyde hydrazones in photosystem **51** with NDIs occurred with an excellent 75–95% yield. Moreover, the yield of stack exchange was nearly independent of the thickness of the photosystem. Control experiments revealed that in the case of initiators without extra NDI templates, the yield drops to 40% for thin photosystems and further decreases with increasing thickness to an irrelevant 25%. This significant difference demonstrates the central importance of templated synthesis for successful stack exchange.

To engineer antiparallel gradients into the red stack of photosystem **53**, partial stack exchange was envisioned. A part of the red cNDI stack, *l*, was removed by brief treatment with hydroxylamine. The produced, shallower holes in photosystem **54** were filled with cNDI aldehyde **6**. The desired photosystem **1** with antiparallel redox gradients in coaxial hole- and electron-transporting channels was obtained.

### Graphical summary of complex transformations

**Scheme 6:**
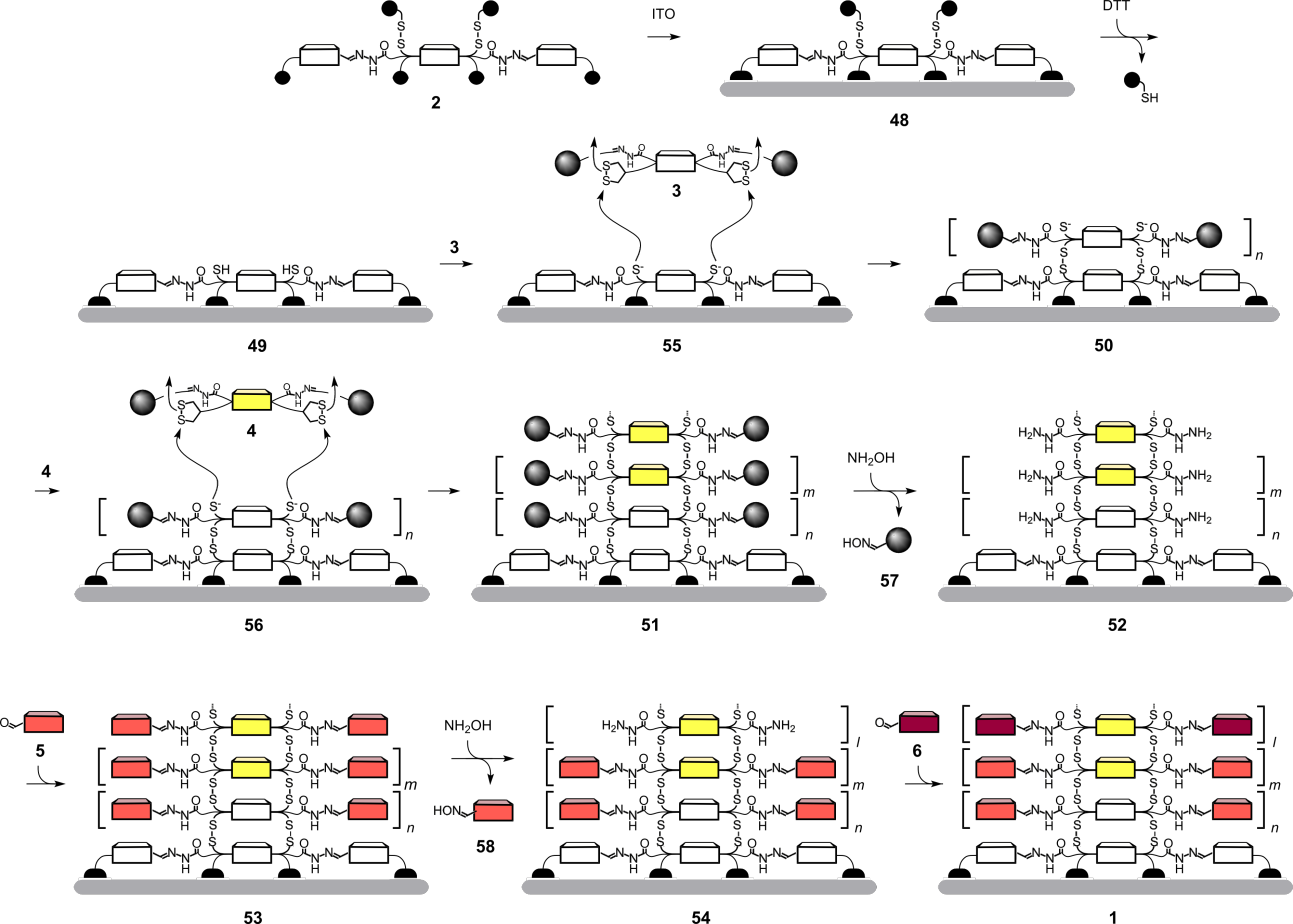
Schematic overview over SOSIP and stack exchange.

Both SOSIP and post-SOSIP stack exchange can be quite complicated to follow in complete molecular structures (Scheme 4 and Scheme 5). We thus summarize both processes in schematic form (Scheme 6). To recapitulate briefly from this perspective, we repeat that the solid-phase synthesis begins with the deposition of initiator **2** on ITO. Activation of monolayer **48** with DTT produces monolayer **49** with free thiols on the surface. Recognition of propagators **3** on the surface of **49** places the strained disulfides of asparagusic acid right on top of the activated thiolates on the surface. Covalent capture by ring-opening disulfide exchange generates new thiolates on the surface of the growing photosystem **55** for continuing SOSIP. The obtained ladderphane **50** is then treated with propagator **4**. Ring-opening disulfide exchange SOSIP via intermediates **56** leads to photosystem **51** with a two-component redox gradient in the π-stack.

Post-SOSIP stack exchange is then initiated by benzaldehyde removal as oxime **57**. The giant pores drilled into photosystem **52** are first filled completely with red cNDI **5**. Subsequently, the partial removal of the new stack in photosystem **53** as oxime **58** and covalent capture of **6** in the more shallow pores in photosystem **54** affords the desired double-gradient photosystem **1**.

## Conclusion

The objective of this brief highlight was to exemplify the synthetic efforts that are often hidden behind short papers on functional systems. Quite extensive multistep synthesis has been covered, followed by innovative surface-initiated polymerization and chemoorthogonal dynamic covalent chemistry. The excellent properties obtained confirm that significant synthetic efforts to build more sophisticated functional systems can be justified and rewarding. In this research, multistep organic synthesis is the means rather than the end. Therefore, the main difference from research dedicated to synthetic methodology is that the individual steps often remain unoptimized as long as the level reached is sufficient to produce large enough amounts of the target molecule without extensive effort and cost. However, the quality, timeliness and beauty of the transformations employed are the same, as is the pleasure of occasional contributions to improve or innovate in the field of organic synthesis.
